# Causes of death contributions to the sex gaps in life expectancy: evidence by education levels from Australia

**DOI:** 10.1186/s12889-026-26888-2

**Published:** 2026-03-26

**Authors:** Wen Su, Jennifer Welsh, Vladimir Canudas-Romo

**Affiliations:** 1 Leverhulme Centre for Demographic Science, Nuffield Department of Population Health, University of Oxford, Oxford, UK; 2 Nuffield College, University of Oxford, Oxford, UK; 3 School of Demography, College of Arts and Social Sciences, Australian National University, Canberra, Australia; 4 National Centre for Epidemiology and Population Health, Australian National University, Canberra, Australia

**Keywords:** Sex differences in mortality, Life expectancy gap, Educational inequalities, Socioeconomic position, Cause-specific mortality

## Abstract

**Background:**

In all high-income countries, including Australia, females have a longer life expectancy than males. The sex gap in life expectancy has been shown to vary according to highest level of education, a measure of socioeconomic position, with a smaller gap among those with higher education. However, the cause contributions to education-related differences in the sex gap in life expectancy remain unclear but may provide insights into the underlying drivers of these inequalities.

**Methods:**

Using Australian data from the 2016 Census of Population and Housing linked to Death Registrations (2016–2019), we quantified the age- and cause-specific contributions to the sex gap in life expectancy in relation to highest attained level of education: university, secondary/post-secondary, and lower than secondary. Date and cause of death information were obtained from Death Registrations using underlying cause and grouped into broad causes of death.

**Results:**

At age 25, the sex gap in life expectancy was 3.79 (3.69, 3.88) years in the total population, and ranged from 2.28 (1.99, 2.56) years among those with a university degree to 4.67 (4.46, 4.89) years among those with lower than secondary education. Across all education groups, the largest contributors to the sex gap in life expectancy were cardiovascular diseases, cancers, and external causes, together accounting for 70–80% of the gap. Although absolute cause of death contributions among those with a university education were smaller when compared to those with lower levels of education, relative contributions from cardiovascular diseases and non-lung cancers were larger among the university educated population. Age-specific contributions were concentrated at older ages for those with a university degree compared to those with lower levels of education (assessed with the interquartile range).

**Conclusion:**

Our study reveals that deaths from cardiovascular diseases, cancers and external causes of death make the largest contributions to the sex gap in life expectancy in Australia. Public health actions targeting these causes, particularly if directed towards preventing deaths among men with lower levels of education, are likely to reduce the sex gap in life expectancy, with potential for larger absolute gains in life expectancy among men with lower levels of education.

**Supplementary Information:**

The online version contains supplementary material available at 10.1186/s12889-026-26888-2.

## Introduction

Since 2006, female life expectancy has consistently exceeded male life expectancy globally [1]. For most countries with long and reliable mortality series, sex gaps in life expectancy have consistently decreased since the 1990s. In recent decades, sex gaps in life expectancy have ranged from 3 years (the Netherlands) [2] to as large as 10 years (e.g. Russia, Ukraine, Belarus, etc.) [3]. Similar to other Western countries, the sex gap in life expectancy in Australia has decreased [2], and was 3.8 years in 2019. Decomposing and quantifying the cause-contributions to these gaps provides insights into underlying health differences between females and males and can be used to support policy and planning decisions [4]. Several studies have decomposed the female advantage in life expectancy, including by age and causes of death. These studies have demonstrated higher mortality rates among males compared to females from external causes (e.g. accidents, and suicides), particularly at younger ages, and non-communicable diseases at older ages (particularly cardiovascular diseases and cancers) [5–8].

Underlying drivers of these mortality differences are theorised to reflect a combination of biological [4, 9] and social factors, including gender norms [10–12]. Females are less susceptible to a number of diseases with an underlying genetic basis because they carry two copies of the X chromosome [13]. They also have a more robust immune system and have lower rates of cardiovascular disease than men at certain ages [14]. Studies have also theorised that the magnitude and historical change in mortality sex differentials are closely linked to sex-specific cigarette smoking patterns [15, 16]. For example, higher smoking prevalence among males compared to females led to excess male mortality during the mid- to late-20th century, particularly from cardiovascular diseases, respiratory diseases, and lung cancers [5, 8]. Additional gender norms are known to shape the exposure to mortality risks [17]. Examples of this include higher risk-taking, a lower tendency to seek preventive care and utilise healthcare resources among males compared to women [18].

Many factors that contribute to socioeconomic inequalities in mortality also shape mortality differences between females and males. Socioeconomic position, such as education levels, operates as a “fundamental cause” that shapes access to resources that reduce the risks and consequences of disease, including in prevention, timely diagnosis, and effective treatment of diseases. Additionally, populations experience different occupational, environmental, and behavioural hazards based on their socioeconomic positions [19]. For instance, those with lower socioeconomic positions, such as lower education levels, may have higher mortality risks compared to their more advantaged counterparts [20]. Socioeconomic inequalities in mortality further exacerbate the differentials in mortality between females and males at the national level [21]. Substantial socioeconomic inequalities in mortality have been demonstrated in high-income countries for both females and males [20, 22]. In Australia, between 2016 and 2017, the gap in life expectancy for those with the lowest compared to the highest levels of education (one measure of socioeconomic position) was 6 years for females and 9 years for males [23]. Previous research has demonstrated subnational differences in the sex gap in life expectancy between those with the highest and lowest education in Australia, with a smaller gap observed among those with the highest (2.6 years) compared to the lowest (6.1 years) level of education [21].

For Australia, the causes of death contributions to these gaps have not been quantified but could provide important insights into the underlying drivers of sex-related disparities in mortality. Such results could provide evidence on how sex- and socioeconomic factors shape exposure to health and mortality risks. The aim of this study was to quantify the cause-contributions to the sex gaps in life expectancy in Australia according to education level using Census data linked to Death Registrations to inform public health interventions that reduce the sex gap in life expectancy.

## Methods

### Data

We conducted analysis of secondary data from the Person Level Integrated Data Asset (hereafter PLIDA), managed by the Australian Bureau of Statistics [24]. The data preparation process followed the methods of Welsh et al. [23, 25]. Briefly, we used individual-level data from the 2016 Census of Population and Housing (hereafter, Census) and Death Registration, linked via the Person Linkage Spine. The Person Linkage Spine is created by combining administrative records from the Medicare Consumer Directory (Australia’s universal health insurer), Data Over Multiple Individual Occurrences (DOMINO, which provides social security information), and Personal Income Tax records, which together result in virtually complete coverage of residents of Australia [26]. The 2016 Census occurred on the night of the 9th of August and had an estimated person response rate of 95.8% [27], and 93% of these records could be linked to the PLIDA Spine. Death Registrations contain a record of all deaths that occurred in Australia and include information on date and underlying cause of death, coded according to the International Classification of Diseases, 10th revision (ICD-10). Among all Death Registration records, 97% were linked to the PLIDA Spine [23, 25]. For this study, we included all deaths occurring among those with a linked Census record from Census night to 31 December 2019. We excluded Census records for temporary visitors and those that did not link to the PLIDA spine. Previous research has shown that Census-Spine linkage does not vary materially by education level [25], and is thus expected to have only a minor impact on the analyses and results. A more detailed account of the data preparation process can be found in Sect. 1 in *Supplementary Material*.

We used the PLIDA combined demographics data to obtain information on sex (female, male) and derived a measure of highest level of education, measured with the Census, grouped into three mutually exclusive categories: university education (bachelor’s degree or higher), secondary/post-secondary (completed secondary school and/ or a post-secondary qualification) and lower than secondary (did not complete secondary school, nor other qualifications). These three education groups corresponded to the 27%, 47% and 26% of the total population, respectively. The equivalence between the education grouping in our analysis and the International Standard Classification of Education can be found in Sect. 1 of *Supplementary Material*.

We measured seven broad causes of death: cardiovascular diseases (CVD), lung cancers, other (non-lung related) cancers, mental (psychiatric) conditions, respiratory diseases, and external causes of death (see Table A1 in *Supplementary Material*). These reflect major cause of death groupings (i.e. CVD, cancers, mental conditions, and external causes), with the addition of lung cancers and respiratory diseases as separate categories. Our decision to include the additional two categories was informed by previous research which found that smoking was a major contributor to sex differences in mortality [5, 8]. Together, the causes of death included in the seven categories account for approximately 90% of all deaths for our data during the period 2016–2019.

### Analysis

For each cause of death, we estimated sex- and education-specific mortality rates by 5-year age group, from age 25–29 to 95 years and older. Mortality rates estimated using the linked Census-Death Registrations data have been shown to be different from official mortality statistics [25], i.e. rates in the linked data were lower at younger ages and slightly higher at older ages compared to those estimated using the unlinked, complete Death Registrations data. If left unadjusted, this would overestimate life expectancy (see supplementary file in [25]). Therefore, we applied mortality rate ratios estimated using linked Census-Death Registrations to mortality rates derived from complete Death Registrations data using estimated resident population counts as the denominator. Further details on the data treatment can be found in Sect. 1 of the *Supplementary Material*. We subsequently smoothed the population and cause of death data into 1-year age intervals using the Penalized-Composite Link Model (PCLM) developed by Rizzi et al. [28].

We constructed life tables with all-cause age-specific death rates by sex and education level using standard life table methods [29]. We generated 95% confidence intervals for female and male life expectancy, as well as for the sex gap in life expectancy, using the life table bootstrap method sampling from binomial distributions [30, 31].

We used demographic decomposition analysis to disaggregate age- and cause-specific contributions to the all-cause sex gap in life expectancy (hereafter referred to as the sex gap in life expectancy) for the total population and according to education level. Demographic decomposition analysis quantifies absolute contributions in terms of years of life attributable to different causes of death across every age, with their sum equalling the all-cause sex gap in life expectancy [32]. The method also quantifies relative contributions as the proportion of contributions from groups of causes with respect to the corresponding all-cause sex gap. 95% confidence intervals for age- and cause-specific contributions are generated using the life table bootstrap method sampling from multinomial distributions [33] (see Sect. 2 in *Supplementary Material*).

We also estimated the modal age contribution, as a measure of central tendency [34, 35], by estimating the age with the highest contribution towards the sex gap in life expectancy, as well as the interquartile range (IQR).

When reporting the cause contributions by age, we partitioned age- and cause-specific contributions into three broad age groups (25–59, 60–84, and 85+) based on noticeable age-patterns (see Fig. 2).

All analyses were performed in R (version 4.4.3) with programming code available online [36].

## Results

For the total Australian population between 2016 and 2019, life expectancy at age 25 years was 60.71 (95% CI: 60.65, 60.78) years for females and 56.92 (56.86, 56.99) for males, representing an all-cause sex gap in life expectancy of 3.79 (3.69, 3.88) years. The largest broad cause contributors to this gap were cardiovascular diseases (1.11 (1.06, 1.15) years, accounting for 30% of the difference), other cancers (0.98 (0.93, 1.02) years, 26%) and external causes (0.77 (0.74, 0.80) years, 21%) (Table 1). Deaths from lung cancer, respiratory disease and other causes made smaller contributions, and deaths from mental conditions were neutral to the sex gap by -0.01 (-0.03, 0.01) years (-1%). 

**Table 1:** Cause of death contributions to the sex gap in life expectancy at age 25 years for the total Australian population and according to highest level of education level, 2016–2019.

Causes	Total		University		Secondary/post-secondary		Lower than secondary	
Contributions	Absolute contribution (years)	Relative contribution (% of all-cause)	Absolute contribution (years)	Relative contribution (% of all-cause)	Absolute contribution (years)	Relative contribution (% of all-cause)	Absolute contribution (years)	Relative contribution (% of all-cause)
All-cause	3.79 (3.69, 3.88)	100%	2.28 (1.99, 2.56)	100%	4.42 (4.26, 4.56)	100%	4.67 (4.46, 4.89)	100%
Cardiovascular diseases	1.11 (1.06, 1.15)	30%	0.82 (0.70, 0.95)	37%	1.28 (1.22, 1.34)	30%	1.28 (1.20, 1.37)	28%
Other cancers	0.98 (0.93, 1.02)	26%	0.59 (0.46, 0.71)	27%	1.18 (1.10, 1.24)	27%	0.97 (0.89, 1.06)	21%
External causes	0.77 (0.74, 0.80)	21%	0.35 (0.28, 0.42)	16%	0.84 (0.80, 0.89)	20%	1.19 (1.11, 1.28)	26%
Lung cancer	0.29 (0.26, 0.31)	8%	0.08 (0.02, 0.13)	4%	0.31 (0.27, 0.35)	8%	0.39 (0.34, 0.44)	9%
Respiratory diseases	0.29 (0.26, 0.32)	8%	0.12 (0.04, 0.19)	6%	0.35 (0.31, 0.39)	8%	0.38 (0.33, 0.44)	9%
Mental conditions	-0.01 (-0.03, 0.01)	-1%	-0.07 (-0.14, 0.00)	-4%	0.05 (0.02, 0.09)	2%	0.05 (0.02, 0.08)	2%
Other causes	0.35 (0.32, 0.38)	10%	0.37 (0.28, 0.45)	17%	0.38 (0.33, 0.42)	9%	0.39 (0.33, 0.45)	9%

Causes of death in the table are arranged by the mean size of the cause-contributions for the total Australian population, from highest to lowest. Values in brackets indicate the 95% confidence interval

**Fig. 1:**
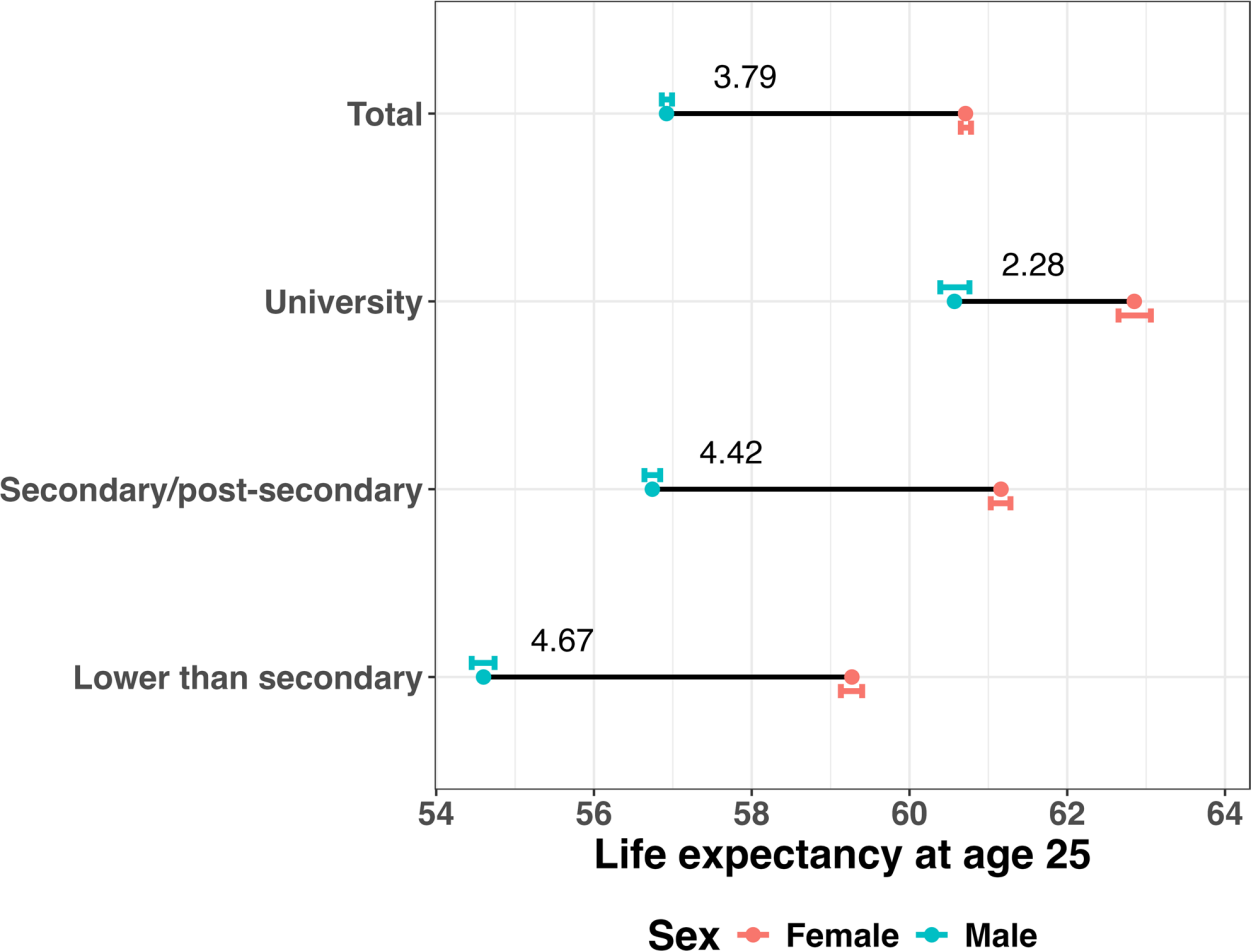
Life expectancy and the all-cause sex gap in life expectancy (in years) at age 25 for the Australian population in total and by highest level of education, 2016–2019 Note: The coloured dot represents life expectancy at age 25 for either females (salmon red) or males (aquamarine). The coloured bar next to the dots represents the 95% confidence interval for life expectancy. The number corresponds to the sex gap in life expectancy (females minus males)

The all-cause sex gap in life expectancy was smaller for those with higher levels of education. The gap was 2.28 years (1.99, 2.56) among those with a university education (female life expectancy: 62.85 (62.65, 63.06), male life expectancy 60.57 (60.39, 60.76)). Compared to those with a university education, the all-cause sex gap was larger for those with a secondary/post-secondary level of education (4.42 years (4.26, 4.56), with female life expectancy: 61.16 (61.03, 61.28), male life expectancy: 56.74 (56.64, 56.84)) and was largest for those with a lower than secondary school education with 4.67 (4.46, 4.89) years (female life expectancy: 59.27 (59.13, 59.40), male life expectancy: 54.60 (54.45, 54.74)), see (Fig. 1).

While the rank order of the cause contributions to the sex gap was broadly similar across education levels, the absolute and relative contributions of each cause varied. Cardiovascular diseases and other (non-lung related) cancers made smaller absolute contributions among those with a university education compared to those with a lower than secondary school education (cardiovascular diseases: 0.82 (0.70, 0.95) vs. 1.28 (1.20, 1.37) years; other cancers 0.59 (0.46, 0.71) vs. 0.97 (0.89, 1.06) years, respectively). However, the relative contribution of these causes was similar or larger among those with a university compared to those with a lower than secondary school education (37% vs. 28%, and 27% vs. 21%, respectively). Contributions from external causes of death were lower in absolute and relative terms among those with a university education compared to those with lower than secondary education (0.35 (0.28, 0.42) vs. 1.19 (1.11, 1.28) years, 16% vs. 26%). The combined contribution from these three causes was similar across levels of education (university 78.4%, secondary/post-secondary 75.1%, lower than secondary 73.9%).

Those with a university education, compared to those with a lower than secondary education, had smaller absolute contributions to the sex gaps from lung cancer (0.08 (0.02, 0.13) vs. 0.39 (0.34, 0.44) years) and respiratory disease (0.12 (0.04, 0.19) vs. 0.38 (0.33, 0.44) years). Relative contributions from these causes of death were also smaller for those with a university education (4% and 6%, respectively) compared to those with a lower than secondary education (9% for both causes). Those with university education had a negative mean absolute contribution from mental conditions (–0.07 (–0.14, 0.00) years), while the other two education levels had positive absolute contributions from this cause (0.05 (0.02, 0.09) years each).

For other causes of death, there were similar absolute contributions to the sex gap in life expectancy for each education level (around 0.38 years). However, the relative contribution was higher for those with a university education compared to lower levels of education (17% compared to 9%).

**Fig. 2:**
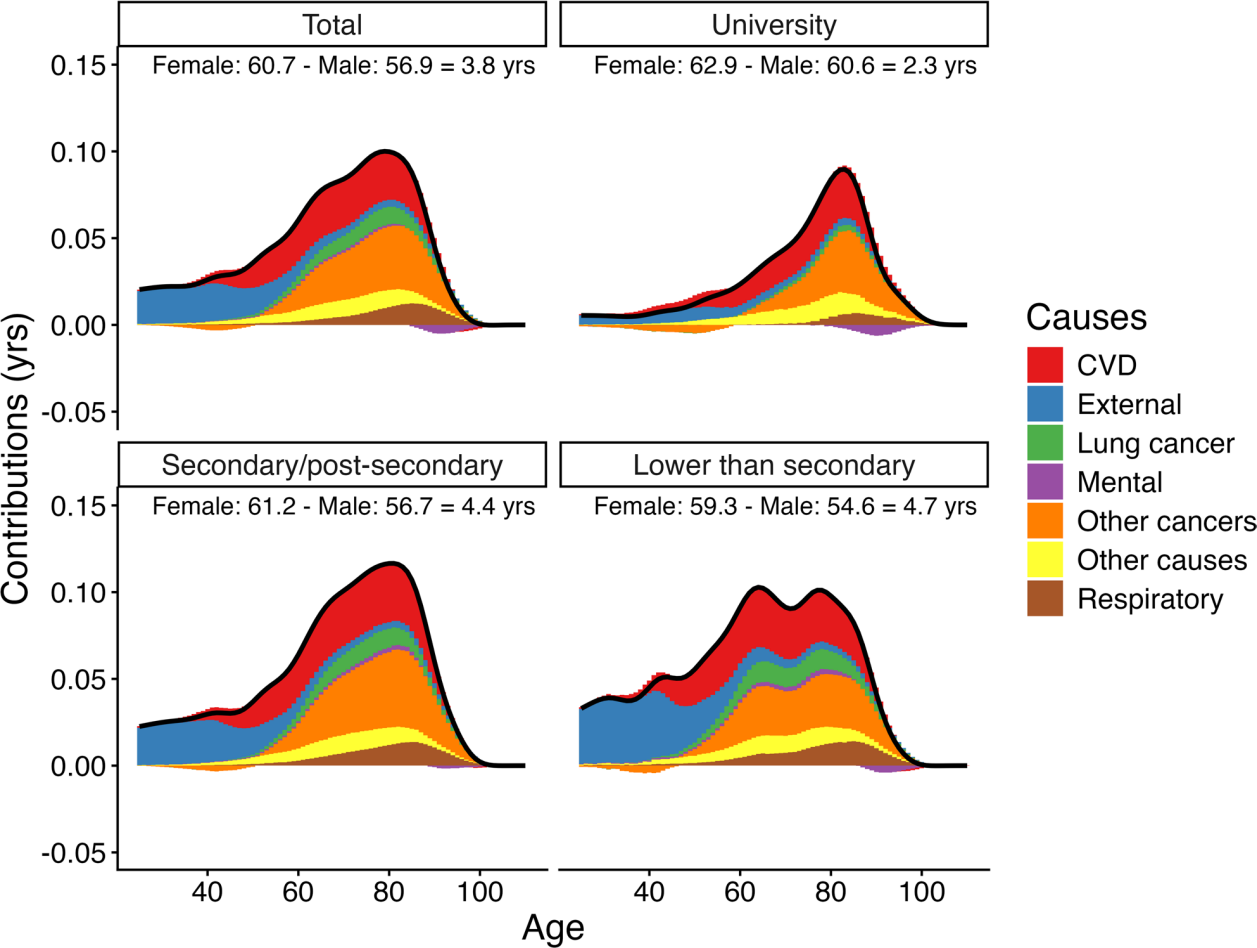
Age- and cause-specific contributions to the sex gap in life expectancy (in years) for the Australian population in total and according to highest level of education, 2016–2019 Note: The dark line represents the age-contribution towards sex gap in life expectancy for each education level and for the total Australian population. Different colours represent different causes of death categories. The cause categories are CVD: cardiovascular diseases, External: external causes, Lung cancer: lung cancer, Mental: mental (psychiatric) conditions, Other cancers: other (non-lung related) cancers, Other causes: other causes, Respiratory: respiratory diseases. We chose to report the cause-specific contributions in age groups (25-59, 60-84, 85+) in the main text for easier interpretability. The first age cut-off (at age 60) is before the first peak age of cardiovascular contribution to the sex gap for those with lower than secondary education, and before mortality from external causes decreases, while the second age cut-off (at age 85) is after the peak in mortality from cardiovascular diseases and other cancers among those with a university education. Results by this age groupings incorporating the confidence intervals can be found in Figure A1 in section 3 of Supplementary Material

Age-contributions to the sex gap in life expectancy (Fig. 2) varied in relation to education level: the modal age contribution was 83 years (IQR:14.9 years) among those with university education and was 81 years for those with secondary/post-secondary education (IQR: 20.3 years). Age-pattern of absolute contributions to the sex gap was bimodal among those with lower than secondary education, with a modal age contribution of 64 years, and a slightly lower second peak at age 78 (IQR: 24.2 years) (see Figure A2 in *Supplementary Material*). For cardiovascular diseases, cancers, and other causes, the modal age contribution was higher, and the IQR smaller, for the population with university education compared to those with lower education levels (see Table A2 in Sect.  3 of *Supplementary Material*).

**Fig. 3:**
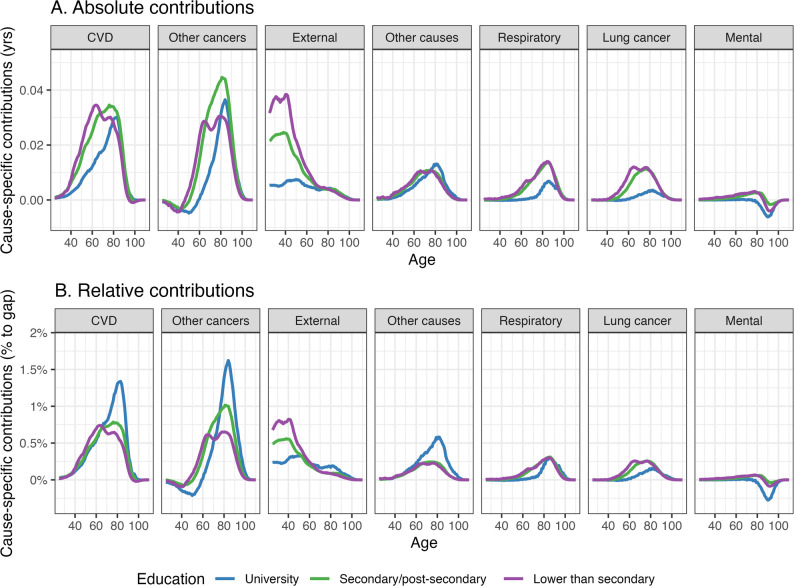
Age- and cause-specific contributions to the life expectancy sex gap according to the highest level of education, Australia 2016–2019 Note: Panel** A** and Panel **B** present respectively the absolute and relative contributions towards sex gaps in life expectancy for each level of education. Absolute contributions are measured in years, while relative contributions are measured as the percentage of contribution to the all-cause sex gap in life expectancy. Different sub-panels within each panel represent different cause categories. The panels are arranged by the scale of average absolute contributions from highest (left) to lowest (right). The cause categories are CVD: cardiovascular diseases, Other cancers: other (non-lung related) cancers, External: external causes, Other causes: other causes, Respiratory: respiratory diseases, Lung cancer: lung cancer, Mental: mental (psychiatric) conditions. Figure A3 and A4 in the Supplementary Material include age- and cause-specific contributions to the life expectancy sex gap with 95% confidence intervals for the absolute and relative contributions, respectively

At younger and working ages (ages 25–59), cardiovascular diseases and external causes made the largest contributions to the sex gap in life expectancy for each education level (Panel A in Fig. 3). The absolute contributions from these two causes were smaller for those with higher levels of education (0.37 (0.33, 0.41) years, compared to 1.01 (0.98, 1.05) years for those with secondary/post-secondary, and 1.46 (1.37, 1.54) years for lower than secondary). The relative contributions showed a similar gradient to the absolute contributions (university 16.5%, secondary/post-secondary 23.0%, lower than secondary 31.2%, see Panel B in Fig. 3). 

Other (non-lung related) cancers contributed negatively (i.e. mortality rates from these causes were higher for females compared to males) from ages 25–59 for all education levels, particularly among those with university education (absolute contribution of − 0.10 (–0.13, − 0.05) years, compared to 0.01 (–0.02, 0.03) for the total Australian population).

Deaths among those aged 60–84 years accounted for the largest proportion of the sex gap in life expectancy across all education levels, but with variation in their relative contributions (university 60.5%, secondary/post-secondary 57.0%, lower than secondary 51.6%). For those with a university education, absolute contributions from this age group were smaller than those with a lower education.

Above the age of 85 years, absolute contributions were similar across different education levels. The negative contributions from mental conditions, that resulted in a male advantage in life expectancy, were largely concentrated within this age group (university − 0.06 (–0.12, − 0.01), secondary/post-secondary − 0.01 (–0.03, 0.01), lower than secondary − 0.04 (–0.05, − 0.02) years).

## Discussion

Our study quantified the age- and cause-specific contributions to the sex gap in life expectancy at age 25 by education level in Australia during the period 2016–2019. Our findings revealed that for each education level, three broad causes of death – cardiovascular diseases, cancers, and external causes – accounted for the majority (70%–80%) of the gap. Although absolute contributions from these causes were smaller among those with higher compared to lower levels of education, the relative contributions were similar or larger for the university educated population. Across all levels of education, the majority of the sex gap in life expectancy reflected deaths at ages 60–84 years. While those with higher levels of education demonstrated age-specific contributions that were compressed at older ages, those with low levels of education had much more variation in the age contributions to the gap, and greater contributions from deaths occurring at younger ages.

All-cause sex gaps in life expectancy were similar between those with a secondary/post-secondary education and a lower than secondary education; gaps for both of these groups were larger than for those with a university education. This similarity reflected comparable contributions among deaths from cardiovascular diseases, cancers and external causes, which is likely related to the fact that these two education groups share similar behaviour-related chronic disease risk factor profiles (e.g., smoking) and exposures to environmental and occupational hazards [37].

We observed a smaller absolute contribution from cardiovascular diseases to the sex gap for those with a university education compared to those with lower education levels, particularly for deaths occurring before the age of 80 years. Further, the unique bimodal distribution of contributions to the sex gap for those with lower than secondary education was, to a degree, a result of higher cardiovascular mortality rates among younger males compared with females. This finding likely reflects a complex interplay between biological differences in cardiovascular disease risk between males and females, and sex- and education-related differences in behaviour-related cardiovascular disease risk factors [5, 38]. Smoking, overweight and obesity, high alcohol intake, physical inactivity and low consumption of fruit and vegetables are more common among males compared to females [39]. While greater proportions of males than females receive guideline recommended treatment following acute cardiovascular events [40], public health strategies that lower the prevalence of chronic disease risk factors may be key to reducing the sex gap in cardiovascular disease mortality (and life expectancy), with larger absolute benefits for those with lower levels of education.

Sex-related inequities in cancer mortality also contribute to the disparity in the sex gap in life expectancy. In addition to sex-related differences in chronic disease risk factors, earlier detection in females, potentially reflecting higher participation in screening programs among females compared to males, is likely to contribute to a female advantage in cancer mortality [41]. Moreover, those with higher education tend to access screening services at a higher rate than those with lower education levels [42]. Promoting males’ participation in cancer screening programs may help to reduce sex-related disparities in cancer mortality, particularly if efforts are directed towards males with lower levels of education.

We observed that deaths from external causes made large contributions to the sex gap in life expectancy, particularly at younger ages, and the sex differences were larger among those with lower levels of education. This is likely a reflection of a combination of factors, including differences in exposure to occupational hazards (manual labour jobs) [43], self-harm [44], and excess risk-taking behaviour [45]. These causes of death are largely considered preventable, highlighting the substantial opportunities to improve life expectancy for males, with larger potential gains among males with lower levels of education [46]. Previous research has noted the potential to reduce injury-related mortality, particularly among males, with strategies such as lowering high-speed limits, raising awareness about dangers of driving while fatigued, and enforcing laws against driving under the influence [47]. Other measures likely to be successful include mental health strategies to reduce suicide rates [48], and policies that promote safe use of alcohol and prescription drugs use to prevent poisoning-related deaths [49].

Lung cancers and respiratory diseases also accounted for a moderate proportion of the sex gap in life expectancy across education levels. Differences in female-male mortality from these causes are likely to reflect, at least in part, higher prevalence of smoking in males than females [50]. In recent years, similar to other high-income countries, the prevalence of smoking has decreased substantially in Australia, but remains higher for males (12.6%) than females (8.7%) overall [39], and for those with lower (15.6%) compared to higher education (5.8%) [51, 52], with larger absolute differences between males and females with lower education levels (31.8% compared to 24.5% among those with lower than secondary education [53]).

Our decomposition results suggest a complex interplay of factors that contribute to sex differences in mortality and socioeconomic inequality. Females and males with higher education levels have greater access to material and non-material resources, which reduce disease risks and improve survival [19]. Consequently, those with higher levels of education have more compressed mortality sex differences at older ages relative to those with lower levels of education. At older ages, larger absolute contributions to the sex gap in life expectancy from cardiovascular diseases, lung cancers, respiratory diseases, and external causes were observed among those with higher compared to lower levels of education. This pattern suggests that education-related gradients in smoking and other behaviour-related risk factors, as well as occupational and environmental exposures, amplify disadvantage for males with lower education levels [15, 16].

Males had lower mortality from non-lung cancer related cancers compared to females, particularly at ages 25–59 years. This pattern has been identified at the national level previously, and reflects relatively high mortality rates from female-specific cancers (e.g. gynaecological, breast) in this age group [54]. Females also had higher mortality rates from mental conditions (which included dementia) at older ages, likely attributable to females living longer on average than males [55, 56].

Other studies have quantified cause of death contribution towards disparities in sex differences in mortality at the subnational level. For example, Sauerberg et al. [18] showed a gradient in sex differences in standardised mortality rates across locations within Europe, with western European regions having smaller sex differences in mortality than regions in eastern Europe. Previous research from the Netherlands and Canada has also found that deaths from cardiovascular disease, lung cancer, and external causes of death make the largest contribution to the sex gap in life expectancy [18, 57, 58]. Our results, using whole-of-population data from Australia, are consistent with these findings from other high-income countries.

Although further narrowing of the sex gap in life expectancy in absolute terms is anticipated in high-income countries, including Australia [35, 59], disparities are expected to persist. At older ages, where most deaths occur, a greater proportion of males have higher levels of education compared to females, and consequently, older males are exposed to lower mortality risks. However, the proportion of females among younger generations obtaining high levels of education has increased substantially in recent years and now exceeds that of males [60] (see Figure A5 in *Supplementary Material*). As a result of this shift, when holding other factors associated constant, these compositional changes in education level between females and males may exacerbate and maintain the disparities in sex gaps in life expectancy in the future [21]. Compositional changes in other factors (morbidity, income, etc.) are also likely to influence this dynamic. Nonetheless, ongoing sex differences in the prevalence of chronic disease risk factors will be a major determinant of sex gap in life expectancy [61] and are amenable to change with public health action.

There are limitations to our study. We used self-reported information on highest level of education collected with the Census. While education level is generally well reported using self-report collection methods [62], a small amount of misclassification is likely. However, given the degree of misclassification is unlikely to differ between females and males, the impact on our conclusions is likely to be minimal [62, 63]. We reported contributions to the sex gaps in life expectancy by categories of causes to understand the broad drivers of sex differences in life expectancy. Further disaggregation of causes may improve our understanding of the dynamics of sex difference in mortality. Such work could also examine the time trends of cause of death contributions to provide further information for targeted public health interventions.

## Conclusion

Overall, our decomposition analyses reveal that the female advantage in life expectancy persisted across all education groups but was consistently smaller at higher levels of education. Sex gaps in life expectancy for each education group were largely driven by cardiovascular diseases, cancers (lung and non-lung related), and external causes, although these causes made larger absolute contributions to the sex gap among lower-educated populations. These findings collectively underscore the importance of addressing sex and education-related inequalities in preventable risks throughout life. This includes tobacco control, injury prevention, and cardiovascular risk reduction, alongside efforts to ensure equitable access to disease prevention, early detection, and effective treatment. 

## Supplementary Information

Supplementary Material 1. Section 1 contains detailed information on data preparation for the analysis. Section 2 comprises a technical explanation on the decomposition method. Section 3 holds figures and tables supplement of the main text.

## Data Availability

Data within the Person Level Integrated Data Asset (PLIDA) is available for approved projects and users: [https://www.abs.gov.au/about/data-services/data-integration/integrated-data/person-level-integrated-data-asset-plida].

## References

[1] Barford A, et al. Life expectancy: women now on top everywhere. BMJ. 2006;332(7545):808. 16601021 10.1136/bmj.332.7545.808 PMC1432200

[2] HMD. Human Mortality Database (HMD). Human Mortality Database 2025 Available from: http://www.mortality.org. Cited 2025 March.

[3] Zarulli V, Kashnitsky I, Vaupel JW. Death rates at specific life stages mold the sex gap in life expectancy. Proc Natl Acad Sci. 2021;118(20):e2010588118. 33972417 10.1073/pnas.2010588118 PMC8157960

[4] Zarulli V, Salinari G. Gender differences in survival across the ages of life: an introduction. Genus. 2024;80(1):10.

[5] Beltrán-Sánchez H, Finch CE, Crimmins EM. Twentieth century surge of excess adult male mortality*.* Proceedings of the National Academy of Sciences. 2015;112(29):8993. 10.1073/pnas.1421942112 PMC4517277 26150507

[6] Case A, Paxson C. Sex differences in morbidity and mortality. Demography. 2005;42(2):189–214. 15986983 10.1353/dem.2005.0011

[7] Pampel FC. Cigarette Use and the Narrowing Sex Differential in Mortality. Popul Dev Rev. 2002;28(1):77–104.

[8] Preston SH, Wang H. Sex mortality differences in The United States: the role of cohort smoking patterns. Demography. 2006;43(4):631–46. 17236538 10.1353/dem.2006.0037

[9] Luy M. Causes of Male Excess Mortality: insights from Cloistered Populations. Popul Dev Rev. 2003;29(4):647–76.

[10] Homan P. Structural Sexism and Health in the United States: a new perspective on health inequality and the gender system. Am Sociol Rev. 2019;84(3):486–516.

[11] Montez JK, Zajacova A, Hayward MD. Explaining inequalities in women’s mortality between U.S. States. SSM - Popul Health. 2016;2:561–71. 27722192 10.1016/j.ssmph.2016.07.004 PMC5049881

[12] Vaidya V, Partha G, Karmakar M. Gender differences in utilization of preventive care services in the United States. J Women’s Health. 2011;21(2):140–5. 10.1089/jwh.2011.2876 22081983

[13] Marais GAB, et al. Sex gap in aging and longevity: can sex chromosomes play a role? Biology Sex Differences. 2018;9(1):33. 10.1186/s13293-018-0181-y PMC6050741 30016998

[14] Viña J, et al. Why females live longer than males: control of longevity by sex hormones. Sci Aging Knowl Environ. 2005;2005(23):pe17–17. 10.1126/sageke.2005.23.pe17 15944465

[15] Lopez AD, Collishaw NE, Piha T. A descriptive model of the cigarette epidemic in developed countries. Tob Control. 1994;3(3):242.

[16] Waldron I. Patterns and causes of gender differences in smoking. Soc Sci Med. 1991;32(9):989–1005. 2047903 10.1016/0277-9536(91)90157-8

[17] Courtenay WH. Constructions of masculinity and their influence on men’s well-being: a theory of gender and health. Soc Sci Med. 2000;5(10):1385–401. 10.1016/s0277-9536(99)00390-1 10741575

[18] Sauerberg M, et al. Sex differences in cause-specific mortality: regional trends in seven European countries, 1996–2019. Eur J Public Health. 2023;33(6):1052–9. 37507140 10.1093/eurpub/ckad111 PMC10710349

[19] Phelan JC, et al. Fundamental causes of social inequalities in mortality: a test of the theory. J Health Soc Behav. 2004;45(3):265–85. 15595507 10.1177/002214650404500303

[20] Mackenbach JP, et al. Determinants of inequalities in life expectancy: an international comparative study of eight risk factors. Lancet Public Health. 2019;4(10):e529–37. 31578987 10.1016/S2468-2667(19)30147-1

[21] Su W, et al. Educational composition effect on the sex gap in life expectancy: a research note based on evidence from Australia. Popul Stud. 2024;78(2):361–9. 10.1080/00324728.2023.2273466 38085530

[22] Yang Y, Kozloski M. Change of sex gaps in total and cause-specific mortality over the life span in the United States. Ann Epidemiol. 2012;22(2):94–103. 22100543 10.1016/j.annepidem.2011.06.006 PMC3337035

[23] Welsh J, et al. Inequalities in life expectancy in Australia according to education level: a whole-of-population record linkage study. Int J Equity Health. 2021;20(1):178. 34344367 10.1186/s12939-021-01513-3 PMC8330008

[24] Australian Bureau of Statistics. Person Level Integrated Data Asset (PLIDA). 2024. Available from: https://www.abs.gov.au/about/data-services/data-integration/integrated-data/person-level-integrated-data-asset-plida.

[25] Welsh J, et al. Education-related inequalities in cause-specific mortality: first estimates for Australia using individual-level linked census and mortality data. Int J Epidemiol. 2021;50(6):1981–94. 10.1093/ije/dyab080 PMC8743133 34999874

[26] Korda RJ, et al. Education inequalities in adult all-cause mortality: first national data for Australia using linked census and mortality data. Int J Epidemiol. 2020;49(2):511–8. 31581296 10.1093/ije/dyz191 PMC7266531

[27] Australian Bureau of Statistics. Census of Population and Housing: Understanding the Census and Census Data, Australia, 2016. 2017. Available from: https://www.abs.gov.au/websitedbs/censushome.nsf/home/2016. Cited 2024 12 Aug.

[28] Rizzi S, Gampe J, Eilers PHC. Efficient estimation of smooth distributions from coarsely grouped data. Am J Epidemiol. 2015;182(2):138–47. 26081676 10.1093/aje/kwv020 PMC4493979

[29] Preston S, Heuveline P, Guillot M. Demography: measuring and modeling population processes. Malden, MA: Blackwell; 2001.

[30] Andreev E, Shkolnikov V. Spreadsheet for calculation of confidence limits for any life table or healthy-life table quantity, in MPIDR Technical Report. 2010, Max Planck Institute for Demographic Research.

[31] Silcocks PBS, Jenner DA, Reza R. Life expectancy as a summary of mortality in a population: statistical considerations and suitability for use by health authorities. J Epidemiol Commun Health. 2001;55(1):38. 10.1136/jech.55.1.38 PMC1731769 11112949

[32] Vaupel JW, Canudas-Romo V. Decomposing change in life expectancy: a bouquet of formulas in honor of Nathan Keyfitz’s 90th birthday. Demography. 2003;40(2):201–16. 12846129 10.1353/dem.2003.0018

[33] Canudas-Romo V, Adair T, Mazzuco S. Cause of death decomposition of cohort survival comparisons. Int J Epidemiol. 2020;49(5):1712–8. 32011680 10.1093/ije/dyz276

[34] Canudas-Romo V. Three measures of longevity: time trends and record values. Demography. 2010;47(2):299–312. 20608098 10.1353/dem.0.0098 PMC3000019

[35] Feraldi A, Zarulli V. Patterns in age and cause of death contribution to the sex gap in life expectancy: a comparison among ten countries. Genus. 2022;78(1):23.

[36] Su W, Welsh J, Canudas-Romo V. Reproducible materials for the manuscript: causes of death contributions to the sex gaps in life expectancy: evidence by education levels from Australia. 2026. Available from: https://osf.io/7pruf/overview?view_only=94189ee8165c4a94accc872cfdaabf02. 10.1186/s12889-026-26888-2 PMC13141274 41888773

[37] Campostrini S, Dal E, Grande, Taylor AW. Increasing gaps in health inequalities related to non-communicable diseases in South Australia; implications towards behavioural risk factor surveillance systems to provide evidence for action. BMC Public Health. 2019;19(1):37. 30621648 10.1186/s12889-018-6323-7 PMC6325833

[38] Khaing W, et al. Effects of education and income on cardiovascular outcomes: a systematic review and meta-analysis. Eur J Prev Cardiol. 2017;24(10):1032–42. 28406328 10.1177/2047487317705916

[39] Australian Bureau of Statistics. National Health Survey, 2022. Canberra, ACT: Australian Bureau of Statistics; 2023.

[40] Australian Institute of Health and Welfare. Use of medications for secondary prevention of cardiovascular disease. ACT: Australian Institute of Health and Welfare Canberra; 2023.

[41] Cook MB, et al. Sex disparities in cancer mortality and survival. Cancer epidemiol biomarkers Prev. 2011;20(8):1629–37. 21750167 10.1158/1055-9965.EPI-11-0246 PMC3153584

[42] Myers L, et al. Understanding the mechanisms underlying the socioeconomic disparities in cancer screening among Australian women. BMC Public Health. 2024;24(1):3437. 39695486 10.1186/s12889-024-20901-2 PMC11653939

[43] Paglione L, et al. Mortality inequalities by occupational status and type of job in men and women: results from the Rome Longitudinal Study. BMJ Open. 2020;10(6):e033776. 32499259 10.1136/bmjopen-2019-033776 PMC7282329

[44] Phillips JA, Hempstead K. Differences in U.S. Suicide Rates by Educational Attainment, 2000–2014. Am J Prev Med. 2017;53(4):e123–30. 28756896 10.1016/j.amepre.2017.04.010

[45] Byrnes JP, Miller DC, Schafer WD. Gender differences in risk taking: a meta-analysis. Psychol Bull. 1999;125(3):367.

[46] Hrzic R, Vogt T. The contribution of avoidable mortality to life expectancy differences and lifespan disparities in the European Union: a population-based study. The Lancet Regional Health – Europe. 2024;46:101042. 10.1016/j.lanepe.2024.101042 PMC11402299 39286330

[47] Chen HY, et al. Fatal crash trends for Australian young drivers 1997–2007: Geographic and socioeconomic differentials. J Saf Res. 2010;41(2):123–8. 10.1016/j.jsr.2009.12.006 20497797

[48] Messias E, et al. Suicide deaths by occupation skill level and educational attainment in the United States. J Occup Health. 2025;67(1):uiae078. 10.1093/joccuh/uiae078 PMC11827085 39727327

[49] Lübker C, Murtin F. Educational inequalities in deaths of despair in 14 OECD countries: a cross-sectional observational study. J Epidemiol Commun Health. 2025;79(2):75–81. 10.1136/jech-2024-222089 PMC11874332 39019490

[50] Australian Institute of Health and, Welfare. Alcohol, tobacco & other drugs in Australia. AIHW: Canberra; 2025.

[51] Banks E, et al. Tobacco smoking and all-cause mortality in a large Australian cohort study: findings from a mature epidemic with current low smoking prevalence. BMC Med. 2015;13(1):38. 25857449 10.1186/s12916-015-0281-z PMC4339244

[52] Aw JYH, et al. Who smokes in Australia? Cross-sectional analysis of Australian Bureau of Statistics survey data, 2017–19. Med J Aust. 2024;220(3):154–63. 38368552 10.5694/mja2.52216

[53] Greenhalgh EM, Scollo MM, Pearce M. Trends over time in smoking among priority populations in Australia, in Tobacco in Australia: Facts and Issues. In: Greenhalgh EM, Scollo MM, Winstanley MH, editors. Melbourne: Cancer Council Victoria; 2024.

[54] Trias-Llimós S, et al. Deciphering the Sex gap in global life expectancy: the impact of female-specific cancers 1990–2019. J Natl Cancer Inst. 2024;JNCI(12):1934–41. 10.1093/jnci/djae191 39141445

[55] Corrada MM, et al. Prevalence of dementia after age 90. Neurology. 2008;71(5):337–43. 18596243 10.1212/01.wnl.0000310773.65918.cd

[56] Letenneur L, et al. Are sex and educational level independent predictors of dementia and Alzheimer’s disease? Incidence data from the PAQUID project. J Neurol Neurosurg Psychiatry. 1999;66(2):177–83. 10071096 10.1136/jnnp.66.2.177 PMC1736218

[57] Spijker J, Poppel Fv, Wissen Lv. Explaining new trends in the gender gap of mortality: Insights from a regional trend-analysis of the Netherlands. Vienna Yearbook of Population Research 2007;2007:61–92.

[58] Trovato F, Lalu NM. Narrowing sex differences in life expectancy: regional variations, 1971–1991. Can Stud Popul. 2001;28(1):89–110.

[59] Glei DA, Horiuchi S. The narrowing sex differential in life expectancy in high-income populations: Effects of differences in the age pattern of mortality. Popul Stud. 2007;61(2):141–59. 10.1080/00324720701331433 17558883

[60] Stoet G. D.C. Geary 2020 Gender differences in the pathways to higher education. Proc Natl Acad Sci 117 25 14073–6. 32513710 10.1073/pnas.2002861117 PMC7322061

[61] Luy M, Wegner-Siegmundt C. The impact of smoking on gender differences in life expectancy: more heterogeneous than often stated. Eur J Pub Health. 2014;25(4):706–10. 25505018 10.1093/eurpub/cku211 PMC4512955

[62] Vo CQ, et al. Validity of self-reported educational level in the Tromsø Study. Scand J Public Health. 2023;51(7):1061–8. 35593433 10.1177/14034948221088004 PMC10599084

[63] Mackenbach J et al. Measuring educational inequalities in mortality statistics. OECD Statistics Working Papers, 2015.

