# Supplementary material for: Causes of death contributions to the sex gaps in life expectancy: evidence by education levels from Australia
Source: BMC Public Health. 2026 Mar 26;26:1467. doi: 10.1186/s12889-026-26888-2 (PMC13141274; doi:10.1186/s12889-026-26888-2)
Supplement: Supplementary file 1 — Supplementary Material 1. Section 1 contains detailed information on data preparation for the analysis. Section 2 comprises a technical explanation on the decomposition method. Section 3 holds figures and tables supplement of the main text. [file 12889_2026_26888_MOESM1_ESM.pdf]

Supplementary Material

Table of Contents

Supplementary Information on Data **2**

Data Preparation 2

Detailed ICD-10 Cause of Death 6

Education levels 6

Decomposition Methods Explained **8**

Supplementary Tables & Figures **10**

1. Supplementary Information on Data

1.1 Data Preparation

We used linked data from Death Registrations and the Census of Population and Housing 2016 (hereafter Census) available via the Person Level Integrated Data Asset (PLIDA) [1], and population statistics relating to the estimated resident population (ERP) [2]. The PLIDA contains individual-level data, linked indirectly via the Person Linkage Spine, which has virtually complete coverage of all Australian residents from 2006 onwards. Death Registrations contain a record of all deaths registered in Australia, including age at death and underlying cause of death, coded according to the International Classification of Diseases, 10th Revision (ICD-10). For this study, final Death Registration files were available for the period 2016-2019. All Australian residents and visitors to Australia on the Census night were in scope for Census 2016, which had an estimated response rate of 95.8% [3]. Death Registrations and Census data were linked to the Person Linkage Spine using deterministic linkage methods, with 97% and 92% linkage rates respectively. Our outcome was cause-specific mortality, ascertained from Death Registrations using underlying cause of death. We grouped causes of death using the ICD-10 codes detailed in Table A1.

Our primary variables of interest were sex (male and female, derived from the combined demographics file within PLIDA) and highest level of education, measured in the Census, and grouped into three mutually exclusive categories: University education (Bachelor's degree or higher), secondary/post-secondary (completed secondary school and/ or a post-secondary qualification) and lower than secondary (did not complete secondary school, no other qualifications). We also measured age, derived from the combined demographics file and based on month and year of birth.

We included people with a Census record aged 25 years and older, and excluded fully imputed Census records, temporary visitors and those whose Census record did not link to the

PLIDA Spine. In our Census study population, there were 1,260 people (<0.001%) without valid information on sex [4], who were excluded from our analysis.

Previous research has demonstrated that mortality rates derived from the linked Census-Death Registrations are different from those in the Australian population (underestimated among young adults, slightly overestimated among older adults [5]). For this reason, we estimated age-, sex- and education-specific mortality rates by applying age group-, sex- and education-specific rate ratios to mortality rates derived from unlinked Death Registrations data (using mid-year estimates of the estimated resident population as the denominator).

We estimated education- and cause-specific mortality rate ratios for each sex and broad age groups (25-44, 45-54, 55-64, 65-74, 75-84, 85+) using negative binomial regression with person-years as the offset. Previous studies by Welsh et al. [5, 6] have demonstrated the validity of using rate ratios to estimate mortality rates with linked Census and Death Registrations data. For each person, person-years were the time between 9 August 2016 (Census night) and date of death or 31 December 2019 (whichever came first).

For each sex and 5-year age group (from 25-29 to 95+), we estimated the average mortality rate for the period 2016-2019 using death counts from the complete (unlinked) Death Registrations data divided by mid-year estimated resident population. Age-, sex-, cause-, and education-specific mortality rates were then derived by applying the mortality rate ratios by broad age group, sex, cause, and education level to the population mortality rates derived for each 5-year age group. A Lexis surface illustration can be found below.

We then fit a Penalized Composite Link Model (PCLM) [see 7] to smooth the data into the shortest age interval possible (1 year) to suit our continuous-time decomposition. The PCLM smoothing assumes the data observed in the coarse age group (in our case the 5-year age group) is an indirect estimate of a latent and finer distribution of data. The PCLM makes modest

assumptions of the underlying data and performs well compared to other smoothing techniques proposed in the past [8].

Our estimates closely resemble comparable estimates derived using the Human Mortality Database (HMD) [9] at the national level for Australia. The difference between our estimates of life expectancy at age 25 and the HMD was around 0.2 years for the Australian national population.

We used a bootstrap method to calculate the 95% confidence interval for the values of life expectancy [10, 11]. We resampled the number of deaths from all-causes 1000 times using a binomial distribution at each age and sex, resulting in 1000 life tables. We then based our calculations of the mean value of the life expectancy and the 95% confidence intervals for life expectancy, as well as those values for the sex gap in life expectancy on these resampled data. Confidence intervals constructed for cause-specific results are mentioned in the following section.

Lexis surface illustration of data coverage

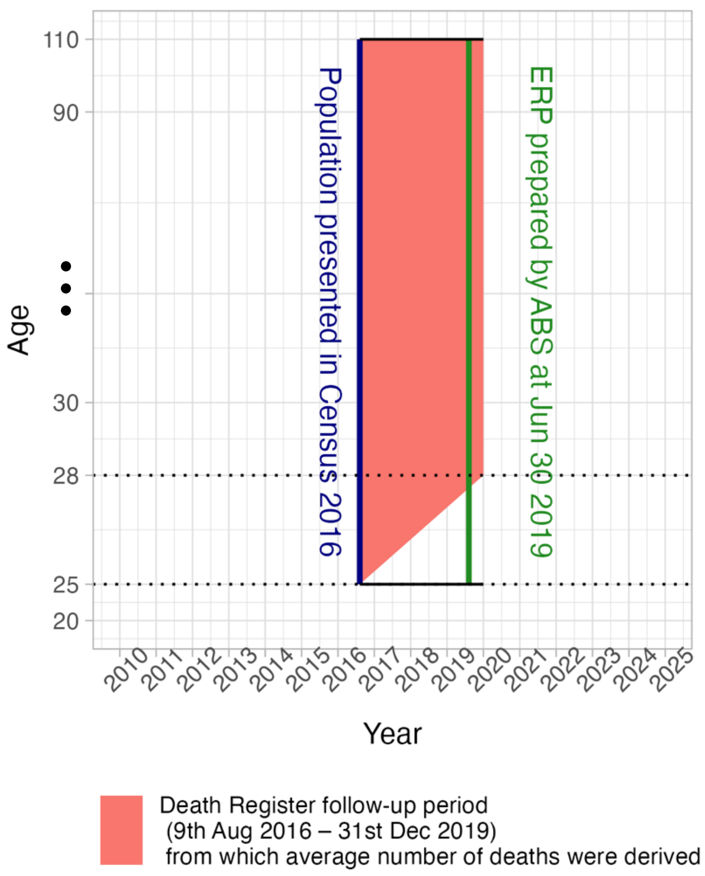

Notes: The bold dark blue line represents the Census study population from which we obtained information on education. The coral pink area represents the period of follow up for population from Census 2016. We calculated an average number of deaths for each age of the Australian population and the age-, sex-, and education-specific mortality rate ratio within this period. The age-specific population mortality rates are calculated by dividing the average number of deaths by the mid-year estimated resident population for each year, estimated by the Australian Bureau of Statistics.

1.2 Education levels

We derived education level from information collected in the Census [1, 6] and grouped them based on International Standard Classification of Education [12] (noting that we used shorter labels throughout the manuscript).

**Australian Census 2016 categorizations**	**International Standard Classification of Education (ISCED)**	**Shorter Labels**
Bachelor's degree or higher (Highest)	ISCED 5–8	University education
Other post-secondary education & finished year 12; Other post-secondary education & didn't finish year 12; No post-secondary education & finished year 12	ISCED 3–4	Secondary/post-secondary
No post-secondary education & didn't finish year 12 (Lowest)	ISCED 0–2	Lower than secondary

1.3 Detailed ICD-10 Cause of Death

Table A1. Causes of death used and their respective International Classification of Diseases codes, 10th Revision (ICD-10).

**Cause Groups**	**Short Name**	**ICD-10 Codes**
Lung (and trachea, bronchus) cancers	Lung cancer	C33, C34
Other cancers	Other cancers	C00-25; C43-C97
Mental (psychiatric) conditions	Mental	F01-F99
Cardiovascular Diseases	CVD	I00-I99
Respiratory Diseases	Respiratory	J30-J98
All External Causes	External	V01–Y89
Other Causes	Other causes	Communicable, maternal, perinatal and nutritional conditions (A00-B99, D50-D53, D64.9, E00-E02, E40-E46, E50-E64, G00-G04, G14, H65-H66, J00-J22, N70-N73, O00-O99, P00-P96, U04, U07.1, U07.2, U09.9, U10.9); Other neoplasms (D00-D48); Diabetes mellitus and endocrine disorders (E10-E14, D55-D64 (minus D64.9), D65-D89, E03-E07, E15-E16, E20-E34, E65-E88); Neuro conditions (G06-G98 (minus G14), U07.0, X41, X42, X44, X45); Sense organ diseases (H00-H61, H68-H93); Digestive diseases (K20-K92); Genitourinary diseases (N00-N64, N75-N98). Skin diseases (L00-L98). Musculoskeletal diseases (M00-M99). Congenital anomalies (Q00-Q99). Oral conditions (K00-K14). Sudden infant death syndrome (R95). Ill-defined diseases (R00-R94, R96-R99)

Source: https://platform.who.int/mortality. We separated lung cancer from other cancers as it is believed that lung cancer has a direct link to smoking behaviours.

2. Decomposition Methods Explained

This decomposition method follows that of Vaupel and Canudas-Romo [13]. We denote life expectancy at age 25 for a specific education level i, or Australian total population, during a given period as $e(25, s, i)$, with the notation s representing sex, either the female or male population. Let a dot • on top of a variable denote the derivative of the variable with respect to sex, i.e. when comparing females to males. The sex gap in life expectancy (female to male) $\dot{e}(25, i)$ for a given education level i is given by:

$$\dot{e}(25, i) = -\int_{25}^{\omega} \ell(x, \bar{s}, i) e(x, \bar{s}, i) \sum_j \dot{m}(x, i, j)\, dx, \qquad \text{(A1)}$$

where the notation ω denotes the highest possible age-group, $\ell(x, \bar{s}, i)$ and $e(x, \bar{s}, i)$ denote the survival function and remaining life expectancy at age x, respectively, for average values between females and males denoted $\bar{s}$. The s is omitted from the variables that denote change, in this case $\dot{e}(25, i)$ and $\dot{m}(x, i, j)$, with the latter representing the age- and cause-specific death rate for age x, the education level i, and the cause of death j.

Let the notation v denote the variable of interest (e.g. $e(x, s, i)$ or $m(x, s, i, j)$). To approximate the derivative with respect to sex for a specific cause i under the continuous change assumption, we use the equations developed in Vaupel and Canudas-Romo [13]:

$$\dot{v}(x, i, j) = \log\left[\frac{v(x, female, i, j)}{v(x, male, i, j)}\right] \left(v(x, female, i, j) v(x, male, i, j)\right)^{\frac{1}{2}}.$$

(A2)

The Vaupel & Canudas-Romo method assumes a constant rate of change in the force of mortality. As a result, it produces smooth age-pattern results, especially when analysing single-year age intervals. The Vaupel & Canudas-Romo method and the traditional Arriaga [14] methods only differ in the assumptions made about changes in the force of mortality. Therefore,

the two methods' results are almost identical, but the Vaupel & Canudas-Romo method is more succinct, since it is in continuous terms.

The confidence intervals for the cause-specific results from the decomposition method are calculated using the life table bootstrap method mentioned in the main text [15]. For each iteration, we sampled the number of deaths from different causes under a multinomial distribution for each age and sex. We then proceed to calculate the corresponding life table and decomposition based on the resampled death counts. We repeat this process 1000 times. In this way, for every iteration we have a complete set of cause-specific decompositions towards the sex gap in life expectancy, preserving the constraints of adding to the total difference.

3. Supplementary Tables & Figures

Table A2. Age with the highest contribution towards sex gap in life expectancy at age 25 and the interquartile range (IQR) of the contributions for the total Australian population and according to highest level of education, 2016–2019

Causes	Australia	University	Secondary/post-secondary	Lower than Secondary
**CVD**	76 (IQR: 19.75)	82 (IQR: 15.95)	77 (IQR: 19.66)	63 (IQR: 20.42)
**Other Cancers**	81 (IQR: 14.08)	83 (IQR: 8.84)	82 (IQR: 14.08)	79 (IQR: 17.11)
**External**	41 (IQR: 19.06)	51 (IQR: 27.86)	39 (IQR: 17.92)	41 (IQR: 16.2)
**Other Causes**	77 (IQR: 17.97)	81 (IQR: 16.49)	77 (IQR: 18.29)	76 (IQR: 19.08)
**Lung Cancer**	78 (IQR: 15.28)	79 (IQR: 12.32)	76 (IQR: 14.8)	65 (IQR: 16.94)
**Respiratory**	86 (IQR: 12.99)	86 (IQR: 9.26)	85 (IQR: 14.45)	85 (IQR: 15.27)
**Mental**	76 (IQR: 2.71)	70 (IQR: 6.24)	81 (IQR: 11.52)	78 (IQR: 12.61)

Notes: *Inter-quantile range is calculated as the shortest age range where 50% of the contributions towards sex gap in life expectancy at age 25 lie. This was also defined in Kannisto [16] as the "C50" index.*

Figure A1. Age-group and cause-specific contributions (in years) to the sex gap in life expectancy for the total Australian population and according to education level, 2016–2019.

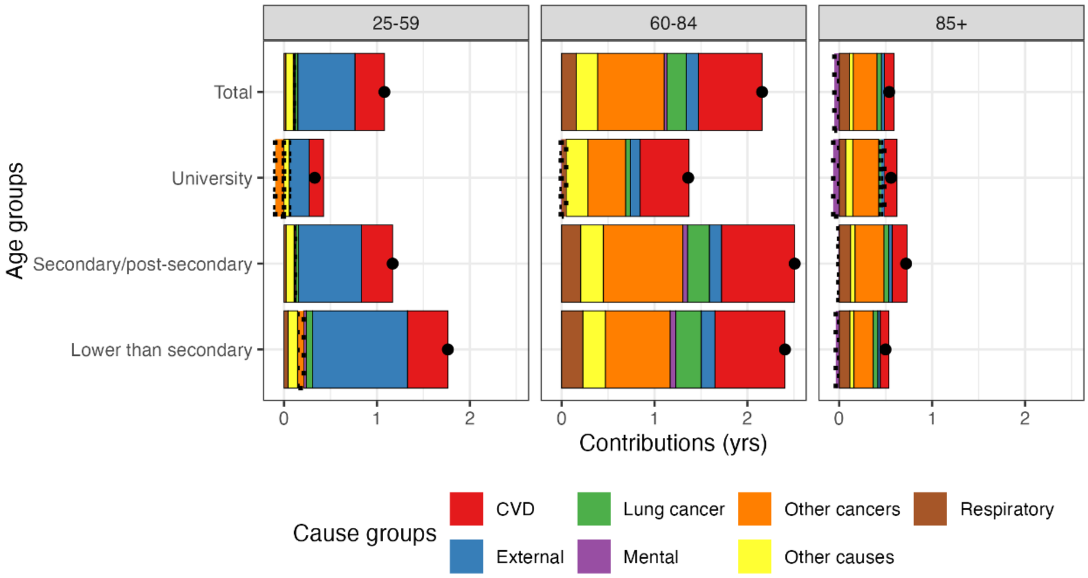

Notes: The component outlined by solid dark lines indicates that the lower bound of the confidence interval for the age group- and cause-specific contributions to the sex gap in life expectancy did not cross zero (statistically significant), while the dotted lines indicate that the lower bound of the confidence interval did cross zero (statistically not significant).

Figure A2. Age-specific contributions to the sex gap in life expectancy (in years) for the Australian population in total and according to highest level of education, 2016–2019.

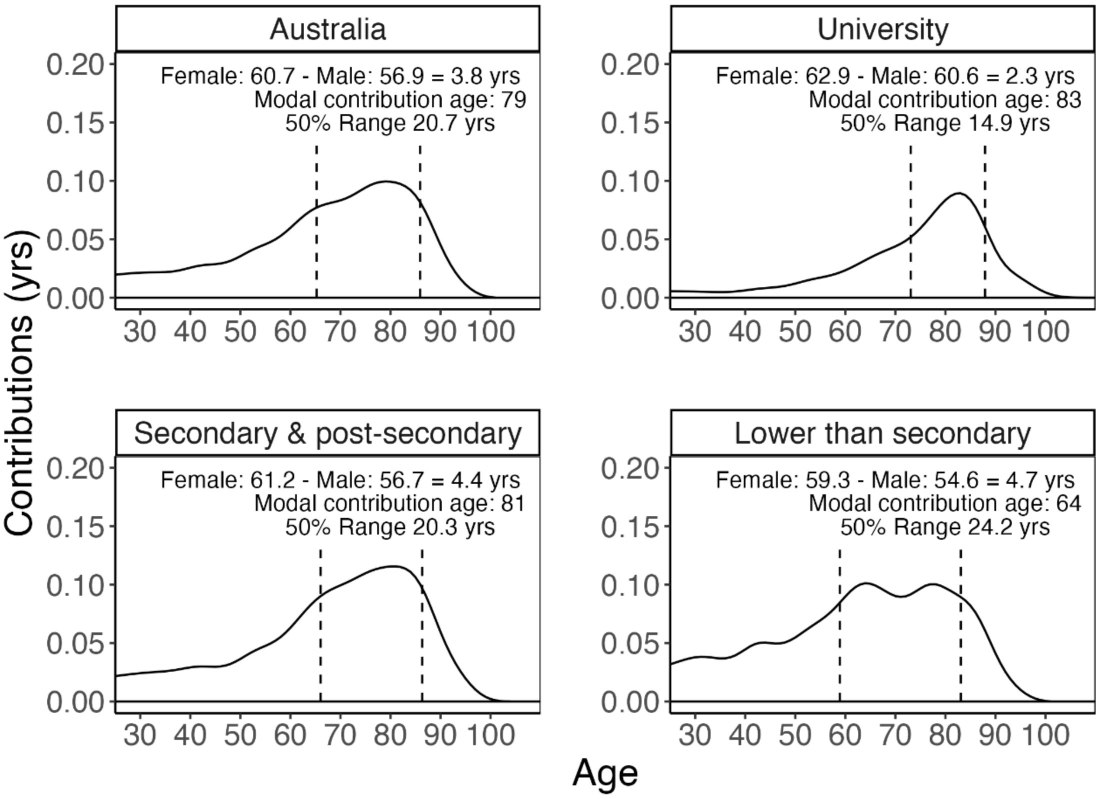

Notes: The first line of text in the caption represents the mean value of the sex gap in life expectancy at age 25 years. The second line represents the modal contribution age, which is the age with the highest contributions towards the gap. The third line represents the interquartile range, i.e. shortest age-interval accounting for 50% of the contribution to the sex gap in life expectancy, which is also indicated with dotted lines on the figures.

Figure A3. Age- and cause-specific contributions (in years) to the sex gap in life expectancy for the total Australian population and for each education level, 2016–2019.

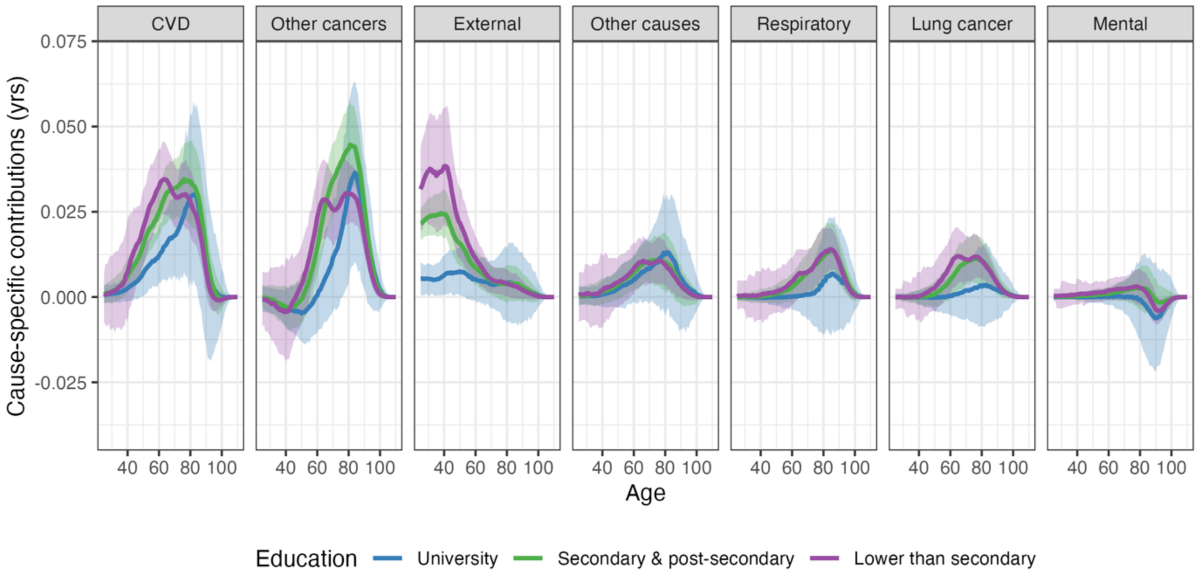

Notes: Each panel includes the mean age- and cause- specific contributions (coloured lines) and 95% confidence intervals of those contributions (coloured bands) to the sex gap in life expectancy for each education level. Different panels represent different cause of death categories.

Figure A4. Relative age- and cause-specific contributions to the sex gap in life expectancy for the total Australian population for each education level, 2016–2019.

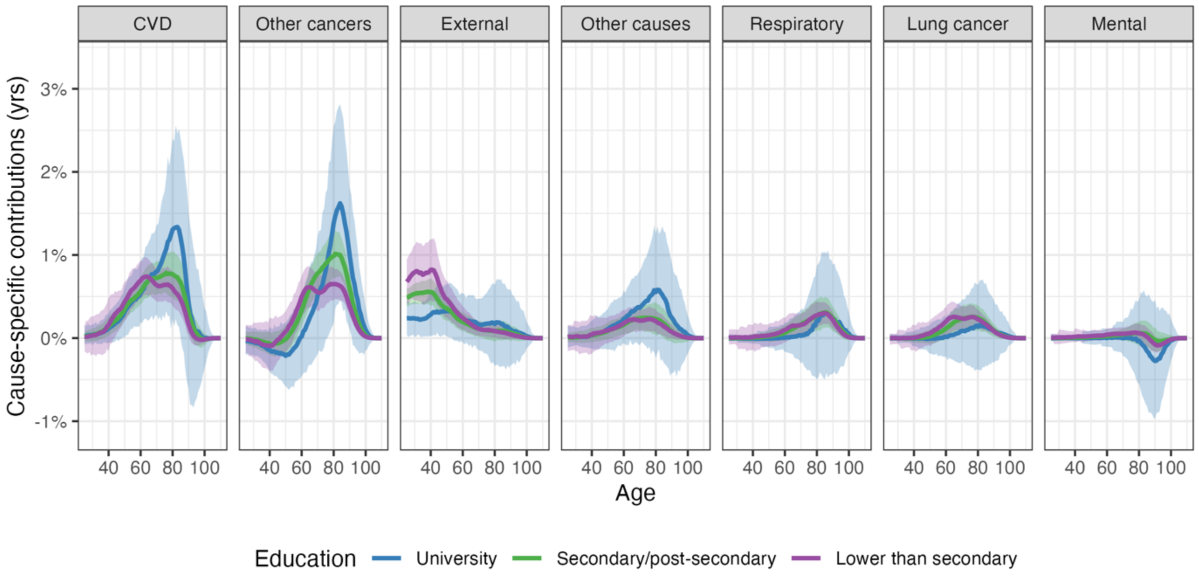

Notes: Each panel includes the mean proportion of each age- and cause-specific contributions (coloured lines), as well as the 95% confidence intervals of those contributions (coloured bands). Different panels represent different cause categories.

Figure A5. Comparison of the distribution of education levels between the Census of 2016 and 2021 for the total Australian population.

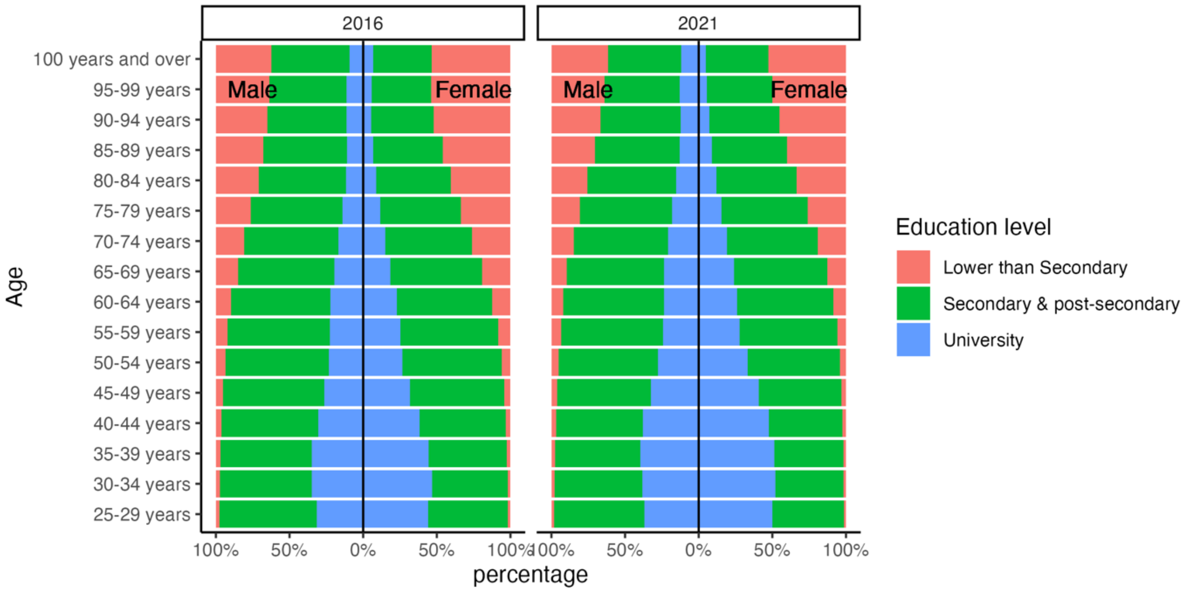

Notes: Each age group sums to 100%. Education composition for males is on the left-hand side of the panel while composition for females is on the right-hand side.

References

1. Australian Bureau of Statistics. *Person Level Integrated Data Asset (PLIDA)*. 2024 13 Jan 2024; Available from: https://www.abs.gov.au/about/data-services/data-integration/integrated-data/person-level-integrated-data-asset-plida.
2. Australian Bureau of Statistics. *National, state and territory population.* 2024 19/09/2024; Available from: https://www.abs.gov.au/statistics/people/population/national-state-and-territory-population/mar-2024#data-downloads-data-cubes.
3. Australian Bureau of Statistics. *Census of Population and Housing: Understanding the Census and Census Data, Australia, 2016.* 2017 [cited 2024 12 Aug]; Available from: https://www.abs.gov.au/websitedbs/censushome.nsf/home/2016.
4. Australian Bureau of Statistics. *Sex And Gender Diversity In The 2016 Census*. Census of Population and Housing: Reflecting Australia - Stories from the Census, 2016 2018 Nov 6 [cited 2022 Jan 23]; Available from: https://www.abs.gov.au/ausstats/abs@.nsf/Lookup/by%20Subject/2071.0~2016~Main%20Features~Sex%20and%20Gender%20Diversity%20in%20the%202016%20Census~100.
5. Welsh, J., et al., *Education-related inequalities in cause-specific mortality: first estimates for Australia using individual-level linked census and mortality data.* International Journal of Epidemiology, 2021. **50**(6): p. 1981-1994.
6. Welsh, J., et al., *Inequalities in life expectancy in Australia according to education level: a whole-of-population record linkage study.* International Journal for Equity in Health, 2021. **20**(1): p. 178.
7. Rizzi, S., J. Gampe, and P.H.C. Eilers, *Efficient Estimation of Smooth Distributions From Coarsely Grouped Data.* American Journal of Epidemiology, 2015. **182**(2): p. 138-147.
8. Rizzi, S., et al., *Comparison of non-parametric methods for ungrouping coarsely aggregated data.* BMC Medical Research Methodology, 2016. **16**(1): p. 59.
9. HMD. *Human Mortality Database(HMD)*. Human Mortality Database 2025 [cited 2025 March]; Available from: Available at: http://www.mortality.org.
10. Andreev, E. and V. Shkolnikov, *Spreadsheet for calculation of confidence limits for any life table or healthy-life table quantity*, in *MPIDR Technical Report*. 2010, Max Planck Institute for Demographic Research.
11. Silcocks, P.B.S., D.A. Jenner, and R. Reza, *Life expectancy as a summary of mortality in a population: statistical considerations and suitability for use by health authorities.* Journal of Epidemiology and Community Health, 2001. **55**(1): p. 38.
12. UNESCO Institute for Statistics, *International Standard Classification of Education ISCED 2011*. 2012, Montreal: United Nations Educational, Scientific, and Cultural Organization.
13. Vaupel, J.W. and V. Canudas-Romo, *Decomposing change in life expectancy: A bouquet of formulas in honor of Nathan Keyfitz's 90th birthday.* Demography, 2003. **40**(2): p. 201-216.
14. Arriaga, E.E., *Measuring and explaining the change in life expectancies.* Demography, 1984. **21**(1): p. 83-96.

15. Canudas-Romo, V., T. Adair, and S. Mazzuco, *Cause of death decomposition of cohort survival comparisons.* International Journal of Epidemiology, 2020. **49**(5): p. 1712–1718.
16. Kannisto, V., *Measuring the compression of mortality.* Demographic Research, 2000. **3**(6).
